# Storm the Capitol: Linking Offline Political Speech and Online Twitter Extra-Representational Participation on QAnon and the January 6 Insurrection

**DOI:** 10.3389/fsoc.2022.876070

**Published:** 2022-05-19

**Authors:** Claire Seungeun Lee, Juan Merizalde, John D. Colautti, Jisun An, Haewoon Kwak

**Affiliations:** ^1^School of Criminology and Justice Studies, University of Massachusetts Lowell, Lowell, MA, United States; ^2^School of Computing and Information Systems, Singapore Management University, Singapore, Singapore

**Keywords:** political participation, online political participation, U.S. Capitol attack, insurrection, Twitter, speech

## Abstract

The transfer of power stemming from the 2020 presidential election occurred during an unprecedented period in United States history. Uncertainty from the COVID-19 pandemic, ongoing societal tensions, and a fragile economy increased societal polarization, exacerbated by the outgoing president's offline rhetoric. As a result, online groups such as QAnon engaged in extra political participation beyond the traditional platforms. This research explores the link between offline political speech and online extra-representational participation by examining Twitter within the context of the January 6 insurrection. Using a mixed-methods approach of quantitative and qualitative thematic analyses, the study combines offline speech information with Twitter data during key speech addresses leading up to the date of the insurrection; exploring the link between Trump's offline speeches and QAnon's hashtags across a 3-day timeframe. We find that links between online extra-representational participation and offline political speech exist. This research illuminates this phenomenon and offers policy implications for the role of online messaging as a tool of political mobilization.

## Introduction

On January 6, 2021, the world watched as rioters stormed the U.S. Capitol—smashing windows, breaking down doors, and sending lawmakers running for their lives—the group intended to stop the count of electoral votes cast in the 2020 U.S. Presidential Election. The breach of the Capitol Building was a moment that revealed deep political divisions and public distrust in democratic institutions and exposed the willingness of some Americans to change the political system through violence forcefully. As law enforcement, politicians, and the media tried to make sense of the attack, images of the event show people displaying assorted insignias affiliated with far-right groups, including those of militias and conspiracy groups.

As of December 2021, more than 700 people from at least forty-four states were arrested for the January 6 insurrection (DOJ, 2021); these individuals vary in age group, gender, and originate from very different geographic communities. Of those arrested, over 300 individuals are alleged to have been involved in violent crimes on Capitol grounds, with more than 200 being accused of assaulting police officers (Hymes et al., 2021); offenses range from weapons possession to kidnapping and murder. Approximately ninety-five percent of those involved are “one-off cases” (Hosenball and Lynch, 2021), which refers to individuals with no prior history of involvement in similar activity. Reflecting on the incident, questions remain regarding the mechanisms that motivated so many people to engage in political violence at the seat of the U.S. government. Given the overwhelming media coverage, we know that the mass of people was there in support of then-president Donald Trump, who previously made several incendiary speeches claiming the election was stolen. Moreover, President Trump publicly urged Vice President Mike Pence and Republican lawmakers not to certify the electoral ballots necessary to legally recognize Joe Biden's election victory.

Based on this context, this current research examines online political discussions that connect the January 6 insurrection to Donald Trump's offline rhetoric. This research is intended to explore the link between offline political language and online extra-representational participation using Twitter within the framework of the insurrection. Integration of a computational method with qualitative thematic analysis was used to understand the ways in which offline influences affect online political engagement. The research asks a set of the following questions: (1) To what extent and how offline speech of Trump is similar to online discourse? And (2) what salient topics were discussed in the online-offline corpora?

To answer these questions, we developed a mixed-methods of computationally informed similarity score and thematic analysis. First, we combined offline speech information with Twitter data during key speech addresses leading up to the Capitol attack. Given the significant size of the data collected and the importance of QAnon in the insurrection (Bond and Neville-Shepard, 2021; Camille, 2021; Crews et al., 2021), we focused on this group as one of the most influential online voices during the incident. Second, we explore the link between Trump's offline speech and QAnon's hashtags across a 3-day timeframe. Third, a similarity score was calculated using the natural language processing-based SentenceTransformer method (Reimers and Gurevych, 2019), a method measuring the parallels between the two sets of corpora and helps to clarify how these offline and online speeches are linked together. Lastly, we use qualitative thematic analysis to examine the topics/themes of the data further. The results demonstrate links between online extra-representational participation and offline political speech.

## Political Communication, Extra-Political Participation, and Online Platforms

### Extra-Political Participation and Political Movements

Our theoretical framework is grounded on extra-representational participation (ERP), resulting from actual or perceived political blockage. Albeit the U.S. has traditionally maintained a clear split between political ideologies, polarization has become far more salient in recent times (Fiorina and Abrams, 2008; Abramowitz and McCoy, 2019; Fermor and Holland, 2020). These friction points have concerned some to speculate about a far greater manifestation of political dissent. After a tumultuous year of protests across most major U.S. cities, Kilcullen (2005) presents a case for what can be considered an incipient American insurgency, defined as a collective movement seeking to overthrow the status quo through the use of subversive tactics, political mobilization, armed conflict, and terrorism (Kilcullen, 2005). To that end, anti-government sentiments and organizational ideologies are central to a wide range of far-right groups (Jones, 2018; Mudde, 2019) that perceive underrepresentation or the loss of power, which are more salient with militias groups (Levitas, 2004; Lyons, 2018; Berlet and Sunshine, 2019). More recent studies link an increase in political violence with the lack of representation in government processes (Daxecker, 2020) and, more specifically, when states accommodate the political elite (Raleigh et al., 2022). Distrust in the government and the sentiment of underrepresentation in the executive processes can lead individuals to engage in ERP. In the United States, specific populations may perceive this loss of power as inevitable, and as such, creating a political sanctuary may insulate themselves from sudden changes throughout the country.

Similarly, the lack of federal-level participation in traditional political processes can have adverse effects, leading to alternative forms of political engagement. Various studies have demonstrated when specific segments of a population are underrepresented in political systems, the likelihood of retribution or violence increases (Gurr, 1993, 2000; Cederman et al., 2010). In most cases, these societal cleavages emerge from ethnic differences and the struggle to gain control of the state. As such, how these segments of populations are represented in executive political systems matters. Whether real or perceived, exclusion from the decision-making processes increases the propensity for political violence or the support of non-state actors challenging the state by those seeking inclusion (Cederman et al., 2010). One example of this dichotomous exclusion condition is Tilly's (1978) polity model, where the system of governance is divided into those who control the state and are privileged to be represented in the executive branch and the challengers seeking to gain political capacity. In order to reach political capacity parity, excluded populations may support ERP, which taps into sub-state networks and non-traditional forms of political mobilization. Kadivar and Ketchley (2018) find that these forms of collective movements are incredibly salient to democratic transitions.

ERP is a way of engaging in the political process that extends beyond traditional forums. For example, the Wall Street Occupy movements that sprung across the globe challenged the governing bodies by engaging in ERP by networking throughout social media platforms (Caren and Gaby, 2011; Adi, 2015). In terms of political capacity, the Occupy movement is well-defined and extends outside an online presence (Costanza-Chock, 2012). This movement challenges the traditional governing system that excluded many citizens from political participation (Della Porta and Reiter, 2012). This form of activism mobilized large protests that criticized representative democratic systems, national infrastructure, or public works (Ramid et al., 2015). Moreover, the Occupy movement sought to engage a broader audience of voiceless individuals under a unified front that targeted political governing bodies. Halvorsen (2012) argues that the Occupy movement initially utilized a decentralized network approach or a do-it-yourself strategy to gain global traction within the youth.

Overall, these movements aim to give a voice to the politically underrepresented; however, it is essential to highlight some of the mechanisms that facilitate ERP. One critical aspect of engaging in ERP is trust in the political systems. Hooghe and Marien (2013) find political trust is positively associated with traditional forms of political participation such as campaign activity or contacting elected officials but negatively associated with ERP. As some early adopters of grievance theory, Craig and Maggiotto (1981) argue there is no reason to become politically active if there is no grievance with the political system. Braun and Hutter (2016) find political distrust and ERP are positively correlated, which aligns with more critical positions that distrust is a strong motivating factor for the underrepresented (Norris, 1999; Rosanvallon, 2008). Similarly, Sika (2020) also finds that countries with constant low political trust and a coercive political system increase ERP probability. The existing literature demonstrates that ERP and underrepresentation are occurrences that affect various political systems. In the same vein, the loss of power or control may also lead to negative feelings by the underrepresented. Petersen (2002) argues individual emotion may play a role in movements that challenge the state. The notion that losing representation in the executive may exacerbate feelings of being dominated by “the other side”. Kemper (1978) argues that social life dynamics of power and status are crucial to societies. Thus, a loss of power and (perceived or actual) representation may lead to a decline in social status and, as a result, a manifestation of negative emotions toward the governing bodies. Likewise, Ha (2017) found the power disparity within Egypt's political representative system led many Coptic Christian populations to feel negatively toward the governing body and the religious majority. Egypt's turmoil during the Arab Spring serves as a vivid example of the power of political mobilization through online mediums. Gerbaudo (2012) highlights the ERP phenomenon through the “choreography of assembly” theory, whereby social media platforms facilitate individuals to politically mobilize and establish “soft” leaders of the movement. Such actions are part of a broader concept of online-offline interactions intertwined within communicative and political spheres. Castells (2015) argues these movements can create “super counter powers” and manifest political accountability against the state.

### Online Communication as Political Participation

Defining interpersonal communication as political participation serves as a point of departure for the current study. Various analyses highlight the influential role of political communication on participatory behavior (Lazarsfeld et al., 1944; Berelson et al., 1954; Stamm, 1985; Viswanath et al., 1990). This active participatory behavior is a fundamental principle of liberal democracies (Pateman, 1970; Schmitter, 1983; Bennett, 1986; Conway, 1991; Crotty, 1991). As a result, through institutionalized participation (i.e., voting or non-traditional processes such as attending a civic forum), local discourse helps reinvigorate democracy (McLeod et al., 1999). Eveland and Scheufele (2000) posit that an individual's concept of the political landscape may be shaped by their mass media consumption and interpersonal communication with others. Similarly, Jung et al. (2011) find that understanding the political landscape and its efficacy are vital mediators for political participation. Carpini and Keeter (1996) argue citizenry depends on mass communication sources for political information that shapes their politics, while some say these interactions can lead to populists politics (KhosraviNik, 2018).

Subsequent studies have provided strong correlations between mass media consumption and political understanding (Chaffee et al., 1994; Weaver and Drew, 1995; Eveland and Scheufele, 2000). Thus, developing a clear picture of the political landscape translates into political participation (Klingemann, 1979; Neuman, 1986; Rosenstone and Hansen, 1993; Verba et al., 1995; Jennings, 1996; Sotirovic and McLeod, 2001; Kaid et al., 2007).

These aspects of interpersonal communication and political participation easily transfer into the online space. Tolbert and Mcneal (2003) found the internet is a powerful mobilizing force for political participation. Their results demonstrate that access to the internet and online political news increased political participation beyond just voting. Similarly, Di Gennaro and Dutton (2006) suggest online activity increases personal efficacy (the belief that they can influence government), and system efficacy (the belief the government is listening to their concerns) is positively significant to political engagement. The online space offers a wide range of platforms users can explore, such as forums, blogs, and social media networks. To that end, Gil De Zúñiga et al. (2009) echo similar findings that online consumption, more poignantly web blogs, is a significant determinant of political participation. Other studies have found online forums can be vital to political involvement. Valenzuela et al. (2012) use social network analysis to support the notion that online discourse is positively correlated with political participation; finding forums that attract like-minded individuals are positively associated with political participation.

Conversely, forums of individuals with different views have less impact on political participation. Finally, Yamamoto et al. (2015) demonstrate the role of social media and mobile platforms on political participation, finding young individuals who consume political information through social media platforms are more likely to engage in political participation—as such, messaging is exceptionally crucial to the mobilization of the underrepresented. Boudreau et al. (2020) argue that ineffective messaging can demobilize specific segments of the target population, and to improve participation, the messenger must strike the proper tone. Vinogradova et al. (2020) argue that some aspects of these communicative elements facilitate rapid and tactical deployment of messaging to generate political mobilization when done correctly. These manifestations of effective communicative efforts can enable the development of political milleus (Krämer et al., 2021).

## The Presence of QAnon and the January 6 Insurrection

One of the most prominent groups capturing attention on January 6 was QAnon, a fringe online conspiracy movement the Federal Bureau of Investigation (FBI) previously labeled as a “dangerous extremist group” (Hughey, 2021; Rubin et al., 2021). The percentage of QAnon supporters charged increases as arrests continue, with estimates reporting QAnon representing more than 8% of arrestees linked to the insurrection (Farivar, 2021). This includes Jacob Anthony Chansley of Arizona (DOJ, 2021), who was famously photographed and became one of the dominant images representative of that day. Chansley wore a horned bearskin headdress, his face painted red, white, and blue, and carried around a flagpole bearing an American flag as he paraded through the Capitol Building; he would later be nicknamed the “QAnon Shaman”. According to court documents, the government alleges Chansley entered the Senate Chamber and left a threatening note for Vice President Pence that said, “It's only a matter of time. Justice is coming.” Among the others arrested are married couples, siblings, parent-and-child(ren) teams, a geophysicist, and a two-time Olympic champion (Fennell, 2021; Feuer, 2021).

In a U.S. Justice Department complaint, the Munn family of Texas accuses a father, mother, and their three children of breaching the Capitol through a broken window. Prosecutors allege Thomas Munn posted on Facebook in late December 2020 he intended to go to Washington for a pro-Trump rally posting, “POTUS HAS REQUESTED YOUR ATTENDANCE WASHINGTON DC JANUARY 6TH 2021.” Later, on January 5, court papers indicate he posted another image showing a homemade sign reading “D.C. Bound We are Q,” referencing QAnon. Doug Jensen, a 41-year-old masonry worker from Des Moines was captured on film wearing a star-spangled “Q” shirt and leading a pack of protesters through the Ohio Clock Corridor threatening Capitol Police Officer Goodman. Jensen took a video of himself showing he was a Trump supporter and professed QAnon believer. Months later, Jensen would admit in court he was fully convinced of the QAnon narrative (Hsu, 2022). Jensen's defense attorney described him as an “intelligent man” who merely “got taken”; asserting Jensen became a “true believer” and was convinced he was doing a noble service by becoming a digital soldier for “Q” (McClatchey, 2021). Although he told the FBI he was “all about the revolution,” there was no evidence supporting him in the planning. “It was a drug. It was absolutely a drug,” said Jitarth Jideja, a former QAnon believer who lost almost 2 years to the movement (Garrett, 2021). These are just a few examples demonstrating how online disinformation resulted in the January 6 offline mobilization.

Unlike many other conspiracy theories, the QAnon narratives gained legitimacy when public officials began to spread disinformation and amplify Q messaging. For example, during the November 2020 election, two QAnon supporters were elected to Congress, Marjorie Taylor Greene won a House seat for Georgia, and Lauren Boebert won a House seat for Colorado. Greene and Boebert were among a dozen Republican candidates who had endorsed or given credibility to QAnon's conspiracies (Tully-McManus, 2020). Later, Democrats would call for the expulsion of Congresswoman Greene for amplifying allegations of election fraud and not discrediting QAnon conspiracy theories (Garrett, 2021). Even President Trump avoided denouncing QAnon by claiming he knew little of the movement despite them being one of his strongest supporters. Coincidently, Michael Flynn, Trump's first national security advisor, posted a 53-s online video depicting oath-taking actions where he recites QAnon phrases and slogans (Cohen, 2020). The video was posted on July 4, 2020, and included the hashtag #TakeTheOath. In addition to these political figures, QAnon has received support from such celebrities as Alex Jones, Curt Schilling, Roseann Barr, Pete Evans, and Eddie Bravo (Crowley, 2020). The acknowledgment of QAnon by these influential public figures enhances the credibility of the conspiracy and appreciably increases the movement's public legitimacy both online and offline.

## Current Study

Social media, particularly Twitter, is an effective communication tool for political communication and mobilization (Gerbaudo, 2012; Enli and Skogerbo, 2013; Sandoval-Almazan and Gil-Garcia, 2013; Theocharis et al., 2015; Lee and Jang, 2021). In the age of Trump, Twitter emerged as his preferred platform to effectively communicate with his supporters and political opponents and convey diplomatic agendas (Ott, 2017; Stolee and Caton, 2018; Simunjak and Caliandro, 2019; Nacos et al., 2021). In addition, Twitter's characteristics, such as brevity, reverse chronological order, and dissemination functionality, facilitated Trump's usage of Twitter as his primary political communication method; which also encompasses Trump's (mis)use of Twitter for delivering messages and mobilizing others after the 2020 election and, in particular, before and after the January 6, 2021 event. This proliferation of misinformation resulted in the closure of Trump's official Twitter account (@realDonaldTrump) on January 8, 2021 (Twitter, 2021), 2 days after the insurrection.

As an exploratory study, this research investigates the link between offline political rhetoric and online ERP by examining Twitter within the context of the January 6 insurrection. This research is part of an ongoing project on political mobilization and the January 6 event. As mentioned earlier, QAnon has been one of the primary catalysts that helped shape extremist discourse and politically mobilize online platforms users. More importantly, the QAnon phenomenon bridged the gap between online and offline spaces, giving its social network non-hierarchical characteristics. Since QAnon is allegedly a loyal supporter of Trump (Bloom and Moskalenko, 2021), and Trump preferred Twitter for his political messaging, we viewed these information sources as a resource to better understand and trace the ERP phenomenon. To that end, we examined tweets and compared them to Donald J. Trump's speech data within a 3-day window by attaining a similarity score between texts through the state-of-the-art natural language processing (NLP) method. We selected a 3-day window to reflect continuity and changes in the dynamic social media discourse.

We hypothesize that online and offline speech are linked together; therefore, if a similarity score between the offline speech and the online corpus is significant, Trump's speech likely resonates well within the Twitter space.

## Data and Methods

### Data Sources and Data Collection Strategy

This research uses a mixed-method framework to integrate computational analysis with qualitative thematic analysis (Braun and Clarke, 2006). As studied by Murthy (2017), a mixed-methods approach to Twitter data is highly beneficial as it allows for reductive research, a type of abductive method that approaches a sense of openness toward one's data and research questions. Murthy argues traditional approaches can be useful, but alternative approaches, such as reduction and grounded theory, have tremendous value to studies of Twitter (Murthy, 2017). Computationally calculated sentence similarity scores based on speech corpus and tweet similarity offer us a quantitative understanding of large-scale data. As text data is inherently qualitative, it is meaningful, and it is imperative to pay attention to its context to understand its meaning. Qualitative modes of inquiry and knowledge of the data are essential to have a more comprehensive understanding. The thematic analysis allows us to inductively derive and explore themes within a more significant event (Andreotta et al., 2019). This approach offers a more nuanced understanding of the underlying connection between offline political rhetoric and online extra-representational participation using Twitter in connection to the January 6 insurrection. This integrated approach assists us in realizing a profound and sophisticated understanding of text data that might not always be discovered purely by relying on calculated similarity scores. Thematic analysis can produce trustworthy and insightful findings (Braun and Clarke, 2006).

We utilized data sources derived from offline and online spaces to accomplish this task. The offline data was taken from our larger dataset focusing on Donald Trump's speeches emerging from rallies, debates, interviews, and significant political topics ranging from early voting to claims of a stolen election. This research focused exclusively on three speeches President Trump delivered shortly before the January 6 insurrection. The speeches in our analysis include January 4, 2021, producing 1,053 sentences; a rally speech at Capitol on January 6, 2021, producing 926 sentences; and a subsequent January 6, 2021, evening speech that produced 19 sentences.

Twitter is one of the most popular and essential online platforms to communicate with others, and as of the second quarter of 2021, it is estimated to have 206 million daily users on its platform, with 38 million emerging from the U.S. (Statistica, 2021). Given its reach, Twitter is used to discuss ideas, shape individual behavior, and even mobilize people for a greater cause (Schill and Hendricks, 2016). In view of its communication role, our online data was pooled from our larger dataset focusing on Twitter activity, a strategy to help us understand how different spaces (i.e., online and offline) may be related to each other.

Our research in this area was in place before the event on January 6, 2021. We had already employed a Twitter search API to collect tweets of certain groups between November 6, 2020, to January 21, 2021, refining our primary dataset to identify QAnon-related hashtags (see Table 1) for this study. Our pre-process of the data involved a restriction to English tweets, removing URLs, mentions, stop words, emoticons, symbols, and punctuations. As a result, the pre-processed data for all hashtags are 5,623,481 tweets (see Table 1A), and the three hashtags included in our study are 1,499,121 tweets (see Table 1).

**Table 1:** Descriptive statistics: similarity scores comparing offline speech and tweets.

	**Offline speech**								
	**January 4**			**January 6, Rally**			**January 6, Evening**		
**Twitter hashtag**	**Mean**	**Max**	**Min**	**Mean**	**Max**	**Min**	**Mean**	**Max**	**Min**
#trump2020	0.535	0.823	0.346	0.543	0.804	0.312	0.594	0.804	0.488
#MAGA2020	0.461	0.662	0.268	0.471	0.665	0.271	0.511	0.595	0.434
#QAnon	0.438	0.680	0.281	0.472	0.707	0.318	0.512	0.597	0.388

*This table is organized by the mean frequencies of January 4*.

### Analytical Strategy

Previous studies use speech/tweet data and found informative results on people's behaviors, specifically for political mobilization studies (Ayeomoni and Akinkuolere, 2012; Schill and Hendricks, 2016). Some researchers use sentence-level analysis in news and social media to find the meaning of sentences in the medium they were exploring (Almer et al., 2020; Asgari-Chenaghlu et al., 2020). Learning from the existing research, we compared speech data with tweets to obtain a sentence-level similarity score between a set of speech text and tweets. Given the online data's size and noisiness, we selected similarity scores to match the offline data. Each sentence of offline data was used as a unit of analysis for matching each tweet in our online data, a technique to measure a sentence-level similarity score. To that end, we used SentenceTransformer (Reimers and Gurevych, 2019) to map a sentence to a 1,024 dimensional dense vector in an embedding space. We then used the RoBERTa-large pre-trained model, showing state-of-the-art performance in multiple NLP tasks. Then, we computed the cosine similarity of the sentences' representations and considered it the similarity of sentences. Next, we compared the top 20 messages based on the similarity scores between each sentence in offline speeches and online tweets (*N* = 39,960). We used an average similarity score of each dataset as a threshold value of twenty tweets. We focused on sentences with above-average scores among the identified data, identifying them as “key” sentences. Three authors read through the entire sample data and individually generated several themes. We then prepared the initial coding of sentence relevance indicators of online ERP. The themes were compared at speech and tweet levels to reach a list of final themes for further thematic analyses of our data. Subsequently, human coders analyzed the data by identifying the relevance of each speech sentence across all offline speeches to online definitions of ERP.

We then used qualitative thematic analysis to identify each speech's themes, matching them with tweets previously identified with sentence similarity scores. The qualitative thematic analysis employed the following steps: familiarizing with data, generating initial codes, searching for themes, reviewing themes, defining and naming themes and producing the product/report (Braun and Clarke, 2006; Nowell et al., 2017). This method can generate trustworthy, reliable, and insightful results (Braun and Clarke, 2006; Nowell et al., 2017). Once the focus of the research/data was established, two authors individually read through each speech sentence and coded themes, comparing their results and deciding on the final themes. Intercoder reliability of Cohen's Kappa was 0.79, which is highly moderate (McHugh, 2012).

**Figure 1:**
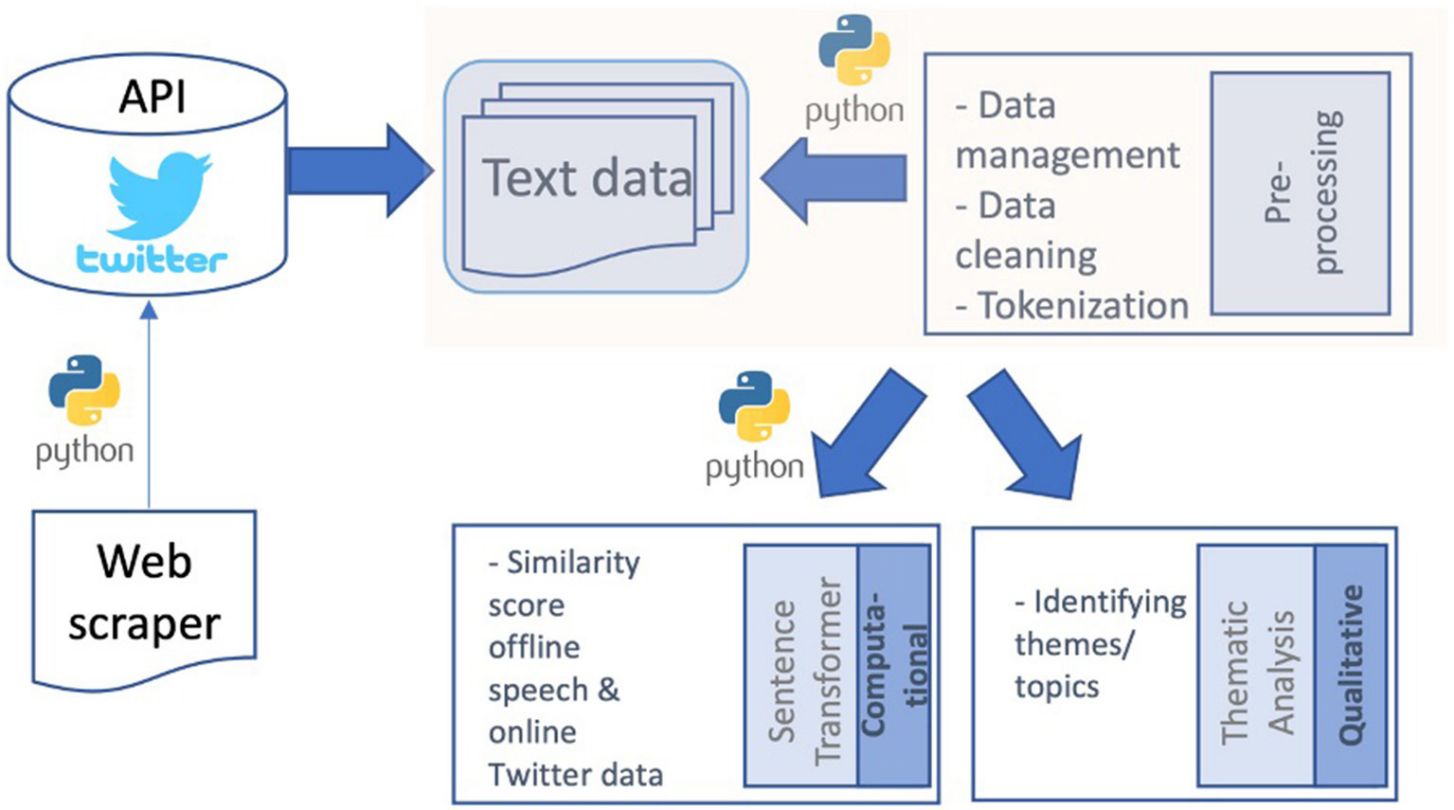
Research procedure.

Figure 1 presents the study's research procedures.

### Descriptive Statistics: Similarity Scores Between Offline Speech and Tweets

Table 1 presents descriptive statistics of three hashtags (#trump2020, #MAGA2020, and #QAnon) that is the current study's focus, while the table also shows the scope of our larger project with 15 different hashtags based on our keywords of specific political groups in the U.S. and their relationship with the speech data. Computationally informed similarity scores comparing offline speech and tweets of each hashtag were presented. The three hashtags were consistently featured as data with the top three averages across the 3 days. Consequently, we created three sub-datasets for the top averaged hashtags for further analyses.

## Results

Qualitative thematic analysis is used to understand the various themes and topics in speech-tweet paired data, generating 3–4 themes and topics per day. In this section, we present core themes of speech-tweet pairs by day. We first identify themes within speech sentences, then use the information to juxtapose them against tweet data themes. On the speech front, Trump's speech throughout the 3-day timeframe before the insurrection has consistent themes such as praising Trump's presidency, persuading Mike Pence to correct the election results, and alleging the 2020 election was stolen. Examination of online tweets presented a more complicated analysis as some support Trump and/or QAnon sentences were positively correlated, while negative correlations took a critical perspective on how Trump has been misleading people. In what follows, we present results interweaving the speech data with three different core tweet datasets.

### January 4 (Rally) Speech and Twitter Data

**Hashtag: #trump2020**

**Themes: The greatest president, Mike Pence, actionable support**

1. In the #trump2020 data, three central themes connected to Trump, with specific reference to his legacy, were identified.

Theme one, “*Trump as the greatest president.”* This was a common phrase repeated across many of the hashtags supportive of the president. As the runoff election results began to be posted in Georgia, tweets emerged from the opposition,“*Now, when you win just a few bellwether counties, you always win the election (1/4 speech).”* In response, supporters of Trump posted on the day of the insurrection the following:

agrees that “is the GREATEST – President ever! ^**^ DO NOT TRUST on JAN 5. ^**^ Rather focus upon overturning the criminally RIGGED election on Jan 6.” (Score: 0.4, Tweeted on 1/6/2021)^1^

This theme emphasizes Trump as the “greatest President” while referencing a narrative directed toward overturning the results of the perceived illegitimate election on January 6, a storyline that dominated many of Trump's public remarks leading up to this date. On January 6, tweets from both sides of the issue filled the virtual forum (reflective in the sentiment and the exponential increase in salience within the #trump2020 dataset), while those called to action were present on the ground in Washington, D.C.

2. The second theme identified within the January 4 speech analysis is specific references to Vice President, Mike Pence. A speech sentence demonstrative of this context included, “*They announced they don't want to do the America First policy (1/4 speech)”*, Trump claimed that Congress, while also inferring Vice President Pence, does not support the idea of the America First policy, which was one of Trump's key campaign platforms in the runup to the election. The following tweet challenges where Pence's loyalty lies while implying he is not supportive of overturning the election result on January 6.

“Mike Pence not loyal to Trump, claims loyalty to ‘Jesus'.” (Score: 0.4, Tweeted on 1/6/2021).

3. The third theme was rigorously calling for actionable support for Trump. In his January 4 speech, Trump references the success of his rallies and encourages his supporters to continue their support despite the election results. “*We have a rally and thousands and thousands... Honestly, I'll go out on the extreme, there's never been anything like this in the history of our country. (1/4 speech).”* Immediately following this speech, tweets of similar themes to Trump's speech increased in frequency and were associated with a call for action. Tweets of individuals purporting to be “patriots” promoted the narrative of “saving” America by supporting the president during this divisive time, as demonstrated in this tweet:

“Patriots from around the world keep coming to support the Mr. President.” (Score: 0.43, Tweeted on 1/7/2021).

More importantly, a well-known powerful slogan emerged - “Storm the Capitol”, which has later become synonymous with the riotous actions of January 6.

“Storm the Capitol now” (Score: 0.34, Tweeted on 1/7/2021).

**Hashtag: #MAGA2020**

**Theme: Mike Pence**

1. Weeks following the 2020 election results and prior to the congressional certification, Trump contentiously argued he won the presidential election and referenced winning several swing states, which was critical to his version of the election outcome. For example, in his January 4 speech, Trump said, “*I won both of them (1/4 speech).”* Tweets connected to the January 4 speech, under the #MAGA2020 hashtag and the Mike Pence theme, had high similarity scores comparable to the findings in the #trump2020 dataset. For example:

“We The People, Have Ur Back Cuz U Have Ours!” (Score: 0.5, Tweeted on 1/6/2021)”.

This includes the following sentence from Trump's January 4 speech, “*We're not going to let it happen over the pass, and I hope Mike Pence comes through for us, I have to tell you (1/4 speech).”* A matching tweet emphasizes,

“Electoral College Vote Count - Watch Mike Pence make history and secure our republic for!” (Score: 0.5, Tweeted on 1/7/2021).

This tweet highlights the importance of Mike Pence's action not to recognize the electoral votes, a course of action promoted by Trump supporters to maintain the current presidency.

**Hashtag: #QAnon**

**Theme: Critiques of Q, Mike Pence**

1. In our #QAnon dataset, Trump January 4 speech states, “*The radical Democrats are trying to capture Georgia's Senate seats so they can wield unchecked, unrestrained, absolute power over every aspect of your lives (1/4 speech).”* While the speech uses antagonistic language directed toward Democrats, subsequent tweets from the Trump opposition emerge, hoping the insurrection participants get a serious illness, inferring the world would be relieved of these individuals.
  “... American right wing fascist are storming the Capitol... These are the voter of the values party... Please catch covid, electors and elected, and rid the world of your idiocy...” (Score: 0.47, Tweeted on 1/7/2021).
2. Mike Pence themes resurfaced again in the #QAnon data. A previous speech we referenced earlier, “*We're not going to let it happen over the pass, and I hope Mike Pence comes through for us, I have to tell you (1/4 speech)” generates the following response by those who oppose Trump:*
  “Just found this on Parler^2^ (which I monitor to keep abreast of and activity). It's getting scary what these guys are willing to do, hope is prepared for tomorrow because it could get ugly...” (Score: 0.49, Tweeted on 1/6/2021).

### January 6 (Rally) Speech and Twitter Data

**Hashtag: #QAnon**

**Themes: Media, Stolen election, Mike Pence**

1. With the January 6 afternoon speech, we identified several themes. Theme one, corrupt media, fake news, and social media, “*as you know the media is constantly asserted the outrageous lie that there was no evidence of widespread fraud. No, we have a corrupt media. We've amassed overwhelming evidence about a fake election*.” In this regard, Trump mentions the idea of a shadowban (a method related to the deprioritizing of accounts). For example, “*And just like the radical left tries to blacklist you on social media, every time I put out a tweet, even if it's totally correct, totally correct. I get a flag*.” However, some tweets do not support either Trump or QAnon but rather provide commentary about the January 6 insurrection. The following tweet, posted on January 7, the day after the insurrection, with a similarity score of 0.52, is an example:
  “… has laid out this insurrection very clearly for literally months. No one listening?” (Score: 0.52, Tweeted on 1/7/2021).
  Conversely, the following tweet links both a reference to a Trump speech and what QAnon stated online:
  “Q did say President Trump would send Presidential text messages. Banning the President from social media is an excellent excuse to make that a reality. It says, National warning messages issued by the President. Can't be turned off.” (Score: 0.52, Tweeted on 1/8/2021).
2. Theme two on illegal votes and the stolen election was one of the salient themes in both the January 6 rally and the January 4 speeches. In the January 6 rally speech, he said, “*They defrauded us out of a win in Georgia*”. This sentiment was picked up online and protracted in Twitter, for example:
  “We remember! We will never forget” (Score: 0.44, Tweeted on 1/8/2021).
  “These are your heroes too. ‘Our supporters are showing some balls about time rest of including the flakes did the same”' (Score: 0.5, Tweeted on 1/7/2021).
  Theme two is linkable to Trump's speech corpus, “*To use a favorite term that all of you people really came up with, we will stop the steal.”*
3. Theme three is on Mike Pence, which is one of the consistent themes throughout Trump's three speeches. A tweet mentioning insurrection lays out an important issue. In a tweet,
  “if he isn't strongly held accountable more of the same will follow. His army is planning more insurrection on Parler as we speak (Score: 0.46, Tweeted on 1/8/2021).”

**Hashtag: #Trump2020**

**Themes: media, Stolen election, Mike Pence**

1. Concerning the media theme, allegedly, Trump followers support Trump's idea that (social) media shares fake news while imposing a ban on him. In this regard, the following tweet resembles what the former president says:
  “Twitter is suppressing free speech. Twitter has attacked conservatives while letting hate speech from the other side, the socialist (Score: 0.61, Tweeted on 1/7/2021).”
2. With regards to illegal votes/stolen election (in relation to the following sentence, “*To use a favorite term that all of you people really came up with, we will stop the steal*.”)
  “rightful winner keep fighting the corrupt” (Score: 0.63, Tweeted on 1/6/2021).
  “God is in control. All fraud will be exposed!” (Score: 0.59, Tweeted on 1/7/2021).
  “We must HOLD THE LINE, the crimes have been committed and certified. It will [be (sic)] interesting what will come of this in the days ahead. Crooked!” (Score: 0.58, Tweeted on 1/7/2021).
  “Yes! Stop the coup attempt! Stop The Steal!” (Score: 0.58, Tweeted on 1/7/2021).
  These tweets are in line with Trump's accusation that the November 2020 election results were fraudulent. This theme of stolen election was thoroughly presented among the three hashtags in this paper. Using the phrase “hold the line” the Twitter user infers this act is a form of attack.
  Despite a significant number of tweets supporting the Stop the Steal campaign, a number of opposing views were tweeted.
  “Never been so proud of my country as today!!! They try to steal the election and we say NO!!! one day down and now only a few more to go” (Score: 0.59, Tweeted on 1/7/2021).
  “Dear President, Truth must be prevail The Cheaters Must be expose And everyone will know everything And everyone will stand with truth Applied law and order Applied law and order Don't sleep the thieves work at the night” (Score: 0.58, Tweeted on 1/8/2021).
3. Regarding Mike Pence, like other hashtags that were identified for having a Pence theme, the following tweets also demand the Vice President stand by the former President Trump's side.
  “I hope VP Pence comes through for Trump!” (Score: 0.75, Tweeted on 1/6/2021).
  “I hope and pray that Trump wins this!! If not this world is going to pieces.....” (Score: 0.66, Tweeted on 1/7/2021).
  “At this point I am praying that Mike Pence does the right thing and call an audit. If not... the our vote no longer will matter. It will be decided by Big Tech and Big Business who will just buy the electors.” (Score: 0.65, Tweeted on 1/6/2021).
  Taken together, tweets with #Trump2020 had three emergent themes—media, stolen election, and Mike Pence. While these themes are identical to those identified in the #MAGA2020's themes, #Trump2020 tweets, they also have slightly higher similarity scores and greater emphasis on Trump.

**#MAGA2020**

**Themes: Media, Stolen election, Mike Pence**

1. In the #MAGA2020 dataset, three themes were identified. This includes observations consisting of both support and opposition mobilization across tweets in response to these themes. The first theme focuses on the media and its relation to the former president. We also note that Twitter permanently suspended his account on January 8th. The former president made a point to target the media by stating, “*and just like the radical left tries to blacklist you on social media, every time I put out a tweet, even if it's totally correct, totally correct*.” Subsequent tweets demonstrated high similarity scores and resonance.
  “The truth will triumph.” (Score: 0.54, Tweeted on 1/7/2021).
  Although the tweet may be vague and inconclusive as to which side the author resonates with, it remains an important theme to discuss. The Trump administration and re-election campaign maintained several conspiracy theories at the ready whenever a situation required its use. As mentioned earlier, Twitter closed the former president's account on January 8th, but according to their assessment, this decision was based on his action, inciting violence. The decision to permanently suspend his account comes after many other far-right accounts were permanently banned from Twitter.
  However, the former president's sentence also resonates with people who opposed this narrative, for example:
  (A) “Hey„ and any other Trump cultists currently spreading lies about January 6th...you can't blame this one on Anti-fascists. We've seen your tweets, Parler messages, F.B. posts, private messages, etc. So have the FBI, CIA and other members of the I.C.” (Score: 0.49, Tweeted on 1/8/2021).
  Without question, the media played a significant role throughout the Trump presidency. Nonetheless, when Twitter suspended his account, other social networking channels became far more active in mobilization. As a result, Parler, among others, experienced an increase in traffic and also misinformation. Tweet A highlights that individuals are fully aware of such movements across far-right social media platforms, but more importantly, many are mobilizing against such false narratives.
2. The second theme identified within the #MAGA2020 dataset focuses on the consistent “stolen election” narrative. During his speech in Washington, D.C., Donald Trump stated, “*To use a favorite term that all of you people really came up with, we will stop the steal.”* As expected, the resonance and mobilization of this sentence permeated across the spectrum.
  (A) “We won it by a lot. Stop stealing the election” (Score: 0.59, Tweeted on 1/6/2021).
  (B) “You guys cant hide behind that statement anymore. What we saw was the perfect representation of. You psychos have been a stain on our country for years and now you are out. It is beautiful.” (Score: 0.51, Tweeted on 1/8/2021).
  Tweet A's resonance aligns with the support of the stolen election narrative. More specifically, the tweet reiterates a sentiment the Trump administration has mentioned since the election results in November. Using the same “we won it by a lot” not only echoes the former president's own words but maintains the idea of an illegitimate election process. Conversely, tweet B's opposition to the speech sentence illustrates how counter-mobilization occurs. The tweet carries a more complex message than just opposing the stolen election narrative. Indeed, it supports the election outcome by signaling “you guys are out” and the notion this period was a mark in American history.
3. The final theme observed within the #MAGA2020 dataset focuses on Mike Pence and his role in the electoral college vote count. During his speech, Trump stated, “*I hope so because if Mike Pence does the right thing, we win the election.”* The subsequent tweets represent the highest similarity scores that resonated exclusively in support of the statement.
  (A) “Electoral College Vote Count - Watch Mike Pence make history and secure our republic for!” (Score: 0.54, Tweeted on 1/7/2021).
  (B) “Come on MIKE were counting on you!” (Score: 0.53, Tweeted on 1/7/2021).
  (C) “For the love of God and Country MIKE!! Grow some balls and do the right thing.” (Score: 0.50, Tweeted on 1/6/2021).

Tweet A and B overwhelmingly support the former president's message that Mike Pence should “do the right thing” and not certify the electoral college count. This creates a misinformation problem that helps mobilize individuals who believe the message. Specifically, the vice president's position over the electoral college is merely to preside over the Senate's process but not intervene, which is outlined in the 12th Amendment. Tweet A, however, seems to expect the former vice president will somehow overturn the election and fall in line with Trump's request. Tweet C, uses more forceful language and includes a quote with a historical, patriotic theme, “for god and country…” which was popularized by the radio call of the team leader of SEAL Team 6 in the Usama Bin Laden during a raid on 11 May 2011.

Additionally, the 1943 short film starring Ronald Reagan, “For God and Country,” depicts the story of army chaplains during the second World War. Tweet C demands Mike Pence overturn the election using volatile language connected to patriotic language. Threatening language and actions against the vice-president were observable and salient throughout that day.

### January 6 (Evening) Speech and Twitter

**Hashtag: #trump2020**

**Theme: Stolen election**

As expected, the speech given after the insurrection event on January 6 generated a large volume of resonance across the Twitter world. The theme of a stolen election was overwhelmingly identified across the #trump2020 dataset, which demonstrated salient resonance in support of the former president's speech but also against the message. This sentence strongly resonated in our data as Trump exclaimed, “*It was a landslide election, and everyone knows it, especially the other side, but you have to go home now.”* The following are tweets in support of the message with the highest similarity scores.

(A) “THEY STOLE THE ELECTION BUT YOU GO HOME” (Score: 0.71, Tweeted on 1/7/2021).
(B) “is an old psyop to identify dangerous Theorists. It worked, people got whacked or had accidents. It's over. The facts are steal of the” (Score: 0.61, Tweeted on 1/9/2021).
(C) “but on another level go home people” (Score: 0.60, Tweeted on 1/7/2021). (D) “Why we all voted for and you stole the election, you knew you didn't have a chance in hell.” (Score: 0.60, Tweeted on 1/7/2021).

It is also essential to consider the idea of a stolen election was pushed by Trump's re-election campaign early on and well-before the election. As a result, this idea was already in the minds of those in support of the former president. Tweets A and C underscore the notion of a stolen election and urge individuals at the Capitol to cease the violent response. B and D's similarity scores demonstrate resonance but more specifically support the idea of a stolen election. Conversely, the sentence also generated opposition. The following tweets highlight the level of contradiction to the message.

(A) “It's finally done. You've lost” (Score: 0.67, Tweeted on 1/7/2021).
(B) “You brainiacs forget that they just stop, once they are all counted...it never matters who is ahead, at any point other than when ALL the votes are counted &amp; they just stop because there are simply no more to count. Come on simpleton¡‘ (Score: 0.64, Tweeted on 1/6/2021).
(C) “...... I.T.'S TIME.. GAME OVER...” (Score: 0.61, Tweeted on 1/7/2021).
(D) “You're already Fired Stop the mess” (Score: 0.60, Tweeted on 1/7/2021).

Tweets A through D acknowledges the election is complete and a new president has been elected. B underscores the fact that recounted votes all returned the same result in support of the new president. Overall, these high similarity tweets make it clear who the loser was. However, D asks that the former president step in and address the violent crowd of insurrectionists.

**#MAGA2020**

**Theme: Dispersal, stolen election**

1. Within the #MAGA2020 dataset, two glaring themes were identified. The first theme, which is in line with most of the data, addresses the notion of a stolen election. Even during the short speech given on the evening of the 6th, which aimed to reduce the violence, the former president reiterated, “*We had an election that was stolen from us.”* The following tweets outline the support for this message:
  (A) “We won it by a lot. Stop stealing the election” (Score: 0.56, Tweeted on 1/6/2021).
  (B) “It was ingenious when picked. He thereby forged a coalition &amp; unified. Certifying the stolen election Pence now has broken this contract.” (Score: 0.50, Tweeted on 1/8/2021).
  Tweet A solidifies the overarching theme of “the stolen election.” But, more importantly, the speech and the supporting tweet utilized “*we”* to establish othering. This was standard practice by the former president during his entire election campaign, political rallies while in office, and his re-election campaign. It is also important to note that othering is also observed across other state-level and county-level political systems. However, when we consider the totality of the “stolen election” message, along with the consistent othering posture displayed over time, it is evident some individuals mobilized extra-politically.
  Although lower in a similarity score, Tweet B adds another dimension to the stolen election theme. This message supplements the “stolen election” idea and focuses on the culpability of the former vice president to certify illegitimate results. Moreover, the tweet also exclaims that Pence broke a contract. While it is also documented that Trump demanded loyalty from his administration heads, the last portion of the tweet aligns with this framework.
2. The second theme focuses on dispersing violent crowds and evacuating the Capitol grounds. Hours after the insurrection and many pleas from various political figures, the former president finally addressed the violent mob. This sentence, “*But go home and go home at peace”* resonated within the Twitter world, and responses with higher similarity scores highlighted mixed sentiments.
  (A) “Screw screw, you incited this you piece of crap, just go away. Screw all you brainwashed, nut jobs.” (Score: 0.46, Tweeted on 1/7/2021).
  (B) “a message to and You let your lord down today due to your lack of follow-through. You just walked away. The streets of D.C. are now silent. The sun has set on DC. (Score: 0.45, Tweeted on 1/7/2021).
  (C) “I see all the ture patriotic Americans are going home before the police come and crack skulls.” (Score: 0.45, Tweeted on 1/7/2021).

Tweets A and C demonstrate discontent with the former president as the catalyst for the insurrections and the mob involved. Tweet A argues those involved were “brainwashed” into mobilizing for the outgoing president. From QAnon's works, lines can be drawn to cultish behavior within such circles involving behavior modification. Tweet C is far more blatant in displaying their displeasure with the events, questioning the crowd's patriotism, and hinting at cowardice when law enforcement began to regain control of the capitol grounds (McGahan, 2020; Badham, 2021; Rothschild, 2021). Conversely, the sentiment of Tweet B is mainly consistent with disappointment as it taps into the notion that the crowd's inability to stay in the fight capitulated any chances to seize the momentum and overturn the election that day.

**Hashtag: #QAnon**

**Theme: Dispersal, stolen election**

Finally, the #QAnon dataset presents very similar themes as the #MAGA2020 dataset, where dispersal from the Capitol grounds and the idea of a stolen election remain salient. However, it is essential to note the higher similarity scores within this data demonstrate opposition rather than support of the speech.

1. The first theme focuses on the dispersal of insurrectionists from Capitol grounds. The sentence “*But go home and go home at peace”* was instrumental in ending the violence on the Capitol grounds that day. However, unlike the #MAGA2020 data, which demonstrated more support for the spoken sentence, the higher similarity scores showed clear thematic opposition for the #QAnon data.
  “I see all the true patriotic Americans are going home before the police come and crack skulls” (Score: 0.45, Tweeted on 1/7/2021).
  This tweet was posted the next day and scored 0.45 in terms of similarity to the spoken sentence. Yet, it is evident from the message itself the author opposes the previous day's events. Moreover, the language used defines the level of animosity and discontent against the perpetrators of the insurrection by calling them “true patriots” and, in some ways, cowards for not staying when the Capitol police were eventually reinforced.
2. The second theme focuses on the idea of a stolen election and the suspected casting of illegal votes. The former president argued, “*We had an election that was stolen from us.”* This sentence generated the following tweets with the highest similarity scores:
  (A) “Hey He used you to commit a coup! He TRIED to subvert DEMOCRACY! You THOUGHT you stormed for freedom, you stormed for a TREASONOUS COUP!!! HE PLAYED YOU!!!” (Score: 0.54, Tweeted on 1/8/2021).
  (B) “Hey, and folks: Is it starting to sink in that there never was a “plan”? This whole time, you got suckered.” (Score: 0.50, Tweeted on 1/9/2021).
  (C) “Where's the evidence you promised! Is this like the electoral fraud “evidence?” Thus far all proffered (none by you) shows influencers and neo-nazis. Charges should be referred on you for inciting insurrection and obstruction of justice first!” (Score: 0.44, Tweeted on 1/8/2021).

Tweets A and C suggest the violent events that unfolded on January 6 were unlawful. Tweet A posted 2 days after the event, utilizes words such as coup and treason to cement the opposition to the “stolen election” idea. The tweet suggests the former president tricked or “played” those individuals into committing an insurrection more poignantly. Tweet C insists the “stolen election” narrative is unfounded and not supported by evidence of wrongdoing in the voting process. Similarly, the tweet charges that such conduct is unlawful and is punishable by law as an attempt to overthrow the government. Lastly, tweet C references neo-nazism (Nowell et al., 2017), which can be attributed to the abundance of far-right symbols observed within the insurrectionist crowd that included Confederacy flags, an assortment of far-right militia emblems such as the Oath Keepers, and Nazi symbols.

The January 6 evening speech has constellated with two themes—stolen election and dispersal. While the theme of stolen election was present in the earlier speech made at Trump's rally on January 6, the theme of dispersal became more relevant after the January 6 (Evening) speech, in particular with #MAGA2020 and #QAnon.

## Discussion and Conclusions

We combine offline key speech addresses comparing Twitter data leading up to the attack on the Capitol on January 6, 2021. We found links between online ERP and offline political rhetoric. In particular, offline political rhetoric contained in speech content was transposed contextually in online ERP.

The main findings of our study are summarized in Table 2. As themes were primarily identified using speech data, each speech contains a unique set of topics, while some themes recur throughout Trump's speech just before, during, and after the insurrection. Following the speech by Donald Trump on January 4, various hashtag datasets (#trump2020, #MAGA2020, #QAnon) emerged with a common subject of Mike Pence, topics which encouraged adherents of Trump and/or QAnon to post supportive tweets. January 6 (Rally) speech shows unified themes (e.g., media, illegal votes/stolen election, and Mike Pence) across all different hashtags. The “Mike Pence” theme appeared not only in the Tweet datasets concerning the January 4 speech, but its presence continued in the January 6 (Rally) speech. The theme of “stolen election” continues in all three hashtags regarding Trump's January 6 (evening) speech, while “dispersal” is emerging in the #MAGA2020 and #QAnon data.

**Table 2:** Summary of the findings.

	**#trump2020**	**#MAGA2020**	**#QAnon**
**January 4 speech**			
Mike Pence			
The greatest president			
Actionable support			
Critiques of Q			
**January 6 (Rally) speech**			
Media			
Stolen election			
Mike Pence			
**January 6 (evening) speech**			
Stolen election			
Dispersal			

*Blue cells indicate a particular theme emerged in the datasets, while black cells indicate such a theme was not found in the datasets*.

These results confirm the importance of effective communicative efforts to mobilize individuals toward a political goal. Furthermore, the fact the same themes appear across various datasets and at different periods suggests political milieus were successfully developed and maintained throughout the 2020 campaign, which some studies have highlighted before (Krämer et al., 2021). Moreover, the themes unearthed by this study give credence to studies that highlight the effects of social media and online platforms consumption on political participation (Yamamoto et al., 2015) and the potential of increasing populist movements (KhosraviNik, 2018).

More specifically, this study highlights the importance of understanding the power of Twitter and any similar social media platforms as an expediter and facilitator of ERP. The results also demonstrate the sincerity within the Twitter messages and support of misinformation purported by QAnon conspiracies, which Trump regurgitated. The content similarity between the speeches and Twitter responses supports the consumption and propagation of misinformation across the online world, which ultimately manifests as offline political violence. Similar online themes and messages highlighted in this study were seen across the Capitol grounds and resulting in some vivid symbols like a makeshift noose.

Albeit some recent studies examine the events of January 6 (Perkins, 2021), or QAnon's conspiracy theories (Crews et al., 2021), the current study is arguably one of the earliest studies to explore the connection between QAnon, the January 6 insurrection, and political mobilization by using both online and offline corpus. More importantly, this research is a unique study that examines the link between Trump's speech and QAnon's influence on ERP online and across social media platforms in the context of the January 6 insurrection. This research innovatively uses “new” mixed methods of computational and qualitative analyses not common in terrorism, security studies, criminology, and sociology. In particular, the mixed methods were designed to combine computational methods of calculating a similarity between speech and large-scale tweets with thematic analysis to interpret underlying meanings and contexts that might not be feasible by only using the computational method.

This research is not without limitations. First, by exploring online and offline QAnon data patterns during the January 6 insurrection, this research—an exploratory study—provides an understanding of the phenomenon—qualitatively. Second, using human coders to identify and verify computer-assisted and compute-analyzed similarity scores between offline and online data provides a more nuanced context for each key sentence we included in our research; however, contextual information might not always be considered. Third, while SentenceTransformer is the state-of-the-art approach for obtaining a vector representation of a sentence, it is not perfect. Thus, our analytical framework and methodological approach helps to better interpret the text. Similarly, the advancement of the NLP enables us to identify more accurate “links” between offline speeches and online participation. Fourth, this study is primarily concerned with Twitter, one of the most popular public social media platforms, but focuses only on one media source. A more comprehensive study of offline speeches and online participation on other social media platforms can be conducted in the future.

This research provides implications for understanding offline and online political mobilization and the influence of online platforms. Using large-scale social media data to analyze offline-online political mobilization during the January 6 insurrection, this research hopes to advance the existing study of the online impact transferred into offline political violence and radicalization. Sentence-level similarity provides an innovative way of comparing offline political speech with online social media resonance in this study's tweets. This research can facilitate further exploration into how online messaging used on social media platforms are utilized by extremist and conspiracy groups (e.g., 4chan, Gab.ai, Parler) to accelerate individual and/or group real-world actions. Improved understanding of the dynamics and influences between offline and online messaging can play a critical role in policy development and possibly, countering violent extremism strategies.

## Data Availability Statement

The data without user identifiers that support the conclusions of this article will be made available by the authors upon request.

## Author Contributions

CL, JM, and JC contributed to conception and design of the study and wrote the first draft of the manuscript. JA and HK performed the data collection and the first part of the analysis, while CL, JM, and JC performed the second part of the analysis. All authors wrote sections of the manuscript and contributed to manuscript revision, read, and approved the submitted version.

## Conflict of Interest

The authors declare that the research was conducted in the absence of any commercial or financial relationships that could be construed as a potential conflict of interest.

## Publisher's Note

All claims expressed in this article are solely those of the authors and do not necessarily represent those of their affiliated organizations, or those of the publisher, the editors and the reviewers. Any product that may be evaluated in this article, or claim that may be made by its manufacturer, is not guaranteed or endorsed by the publisher.

## Footnotes

^1^Tweets are presented as original throughout this paper. In other words, typos, quotation marks, and capital letters are original. We present each tweet in a bracket (without italic), which indicates its similarity score and a published date that was made public, whereas speech sentences were presented as italic.

^2^Parler is an app which is arguably predominantly used by QAnon and Trump supporters (Nicas and Alba, 2021).

## Appendix

**Table 1A:** Descriptive statistics: similarity scores comparing offline speech and tweets (Full dataset).

	**Offline speech**								
	**January 4**			**January 6, Rally**			**January 6, Evening**		
**Twitter hashtag**	**Mean**	**Max**	**Min**	**Mean**	**Max**	**Min**	**Mean**	**Max**	**Min**
#trump2020	**0.535**	0.823	0.346	**0.543**	0.804	0.312	**0.594**	0.804	0.488
#MAGA2020	**0.461**	0.662	0.268	**0.471**	0.665	0.271	**0.511**	0.595	0.434
#QAnon	**0.438**	0.680	0.281	**0.472**	0.707	0.318	**0.512**	0.597	0.388
#WWG1WGA	0.438	0.709	0.252	0.454	0.719	0.266	0.491	0.601	0.387
#War	0.416	0.709	0.249	0.422	0.664	0.265	0.453	0.520	0.366
#savethechild	0.411	0.681	0.256	0.383	0.587	0.219	0.428	0.520	0.310
#SaveOurChild	0.398	0.701	0.239	0.373	0.569	0.211	0.420	0.498	0.292
#Trumpinsurre	0.394	0.637	0.226	0.450	0.666	0.252	0.498	0.615	0.390
#Q	0.392	0.576	0.258	0.408	0.571	0.267	0.433	0.504	0.346
#QAnons	0.392	0.668	0.219	0.446	0.790	0.272	0.486	0.635	0.376
#thestorm	0.320	0.503	0.168	0.353	0.592	0.202	0.389	0.486	0.302
#Greatawakening	0.298	0.451	0.170	0.311	0.437	0.167	0.332	0.387	0.277
#StormIsUponU	0.215	0.426	0.061	0.309	0.451	0.149	0.336	0.410	0.282
#neonrevolt	0.124	0.241	0.013	0.136	0.235	0.036	0.146	0.223	0.088
#Commiebast	0.121	0.355	-0.101	0.135	0.313	-0.035	0.144	0.286	0.039

*This table is organized by the mean frequencies of January 4. This paper is only focused on the first three hashtags datasets (in yellow)*.
